# Neurodevelopmental Plasticity in Pre- and Postnatal Environmental Interactions: Implications for Psychiatric Disorders from an Evolutionary Perspective

**DOI:** 10.1155/2015/291476

**Published:** 2015-04-30

**Authors:** Young-A Lee, Yoshie Yamaguchi, Yukiori Goto

**Affiliations:** ^1^Department of Food Science & Nutrition, Catholic University of Daegu, Gyeongsan-si, Gyeongbuk 712-702, Republic of Korea; ^2^Primate Research Institute, Kyoto University, Inuyama, Aichi 484-8506, Japan

## Abstract

Psychiatric disorders are disadvantageous behavioral phenotypes in humans. Accordingly, a recent epidemiological study has reported decreased fecundity in patients with psychiatric disorders, such as schizophrenia and autism spectrum disorders. Moreover, the fecundity of the relatives of these patients is not exceedingly higher compared to the fecundity of the relatives of normal subjects. Collectively, the prevalence of psychiatric disorders among humans is expected to decrease over generations. Nevertheless, in reality, the prevalence rates of psychiatric disorders in humans either have been constant over a long period of time or have even increased more recently. Several attempts to explain this fact have been made using biological mechanisms, such as de novo gene mutations or variants, although none of these explanations is fully comprehensive. Here, we propose a hypothesis towards understanding the biological mechanisms of psychiatric disorders from evolutionary perspectives. This hypothesis considers that behavioral phenotypes associated with psychiatric disorders might have emerged in the evolution of organisms as a neurodevelopmental adaptation against adverse environmental conditions associated with stress.

## 1. Introduction

Psychiatric disorders are disadvantageous behavioral phenotypes that are subject to therapeutic treatments in modern human society. A recent epidemiological study has reported decreased fecundity in psychiatric patients [1]. Nevertheless, psychiatric disorders have been present in humans with constant or, recently, increased prevalence, a phenomenon that persists even after discounting for changing diagnostic criteria [2–4]. This raises the question as to why psychiatric disorders that are disadvantageous and lead to decreased reproductive successes in humans have not vanished but have been maintained in the process of evolution.

In regard to the biological mechanisms of psychiatric disorders, two major issues should be taken into consideration. First, although psychiatric disorders are genetic and inheritable, environmental factors are often associated with their pathogenesis. In such environmental factors, “stress” appears to be one of the most important factors. Another issue is “neurodevelopment.” Some psychiatric disorders, such as attention deficit/hyperactivity disorder (ADHD) and autism spectrum disorder (ASD), are childhood onset disorders, and, therefore, the involvement of neurodevelopmental deficits is relatively clear in these disorders. In contrast, the onsets of other psychiatric disorders, such as schizophrenia and major depressive disorder (MDD), occur during adulthood in most cases. Nevertheless, neurodevelopmental deficits, which could occur even before birth, have been implicated even among adult onset psychiatric disorders.

In this paper, we discuss the connections between these two major issues with evolutionary perspectives. This leads to the proposal of a hypothesis that the stress-induced neurodevelopmental changes that occur during the pre- and/or early neonatal periods may be understood to be adaptation against expected postnatal stressful environments, and, therefore, psychiatric disorders associated with such prenatal stress-induced neurodevelopmental changes may have evolved and remained in humans as adaptation strategies against adverse environmental conditions.

## 2. Stress Effects on Brain Function from an Evolutionary Perspective

Stress is an adverse environmental factor that is likely strongly associated with psychiatric disorders. For instance, stress has been shown to be involved in the onset, exacerbation, and precipitation of symptoms in schizophrenia [5] and MDD [6]. Epidemiological studies have reported that antenatal maternal exposure to stress during pregnancy also increases the risk of developing psychiatric disorders, such as schizophrenia, ASD, and ADHD in offspring [7–11].

Consistent with the strong association between stress and psychiatric disorders, stress, especially in the form of a chronic, repeated exposure, has been shown to cause an assortment of brain dysfunctions, including cognitive deficits in the working memory [12], long-term memory [13], and behavioral flexibility [14], as well as affective impairments, such as anhedonia [15], heightened anxiety [16], and fear conditioning [17]. A deficit in long-term memory is one of the most investigated brain dysfunctions which is caused by chronic stress, and this deficit is observed across different species, such as rodents [13], nonhuman primates [18], and humans [19]. Indeed, long-term memory is a brain function that plays a pivotal role in the survival of many organisms as it is required for the forging of foods and avoidance of predators. Thus, a deficit in long-term memory significantly endangers organism survival and reproduction.

When these disadvantageous stress effects on brain function are considered from evolutionary perspectives, several questions, such as those listed below, arise:

1. Research has shown that a similar, if not identical, pattern of stress-induced long-term memory deficit is observed in rodents [13], nonhuman primates [18], and humans [19]. Because the divergence between rodents and primates is estimated to have occurred approximately 60~100 million years ago [20], the biological process that causes stress-induced memory impairment (i.e., neuroplasticity deficit) had already been present in organisms before the divergence of rodents and primates and has been maintained for the past 100 million years, even though it appear to be a “disadvantageous” phenotype that could affect reproductive success.
2. Recent studies have revealed that stress induces behavioral changes and associated gene expressions through epigenetic mechanisms [21]. Moreover, such stress-induced epigenetic changes are inheritable across generations through parental gametes [22–24]. Thus, organisms have become equipped with biological mechanisms that can deliberately transmit stress-induced changes to descendants, with quite atypical, Lamarckian-like mechanism, although stress-induced changes are thought to be “disadvantageous” [25].
3. Epidemiological studies have reported that antenatal maternal stress exposure during pregnancy increases the risk of psychiatric disorders in the offspring. Consistent with these epidemiological investigations, animal studies have shown that prenatal stress causes neurodevelopmental deficits, which in turn disrupt various cognitive and affective functions after birth [26–30]. A fetal brain is more plastic than an adult brain, as evidenced by the greater recovery of fetal brains following insult compared to adult brains [31, 32]. Thus, although a fetal brain is highly plastic and is in an immature state, compensation against stress-induced neurodevelopmental deficits does not seem to take place.

In the following sections, we further discuss these issues, along with specific focuses on (1) the interaction of prenatal and postnatal stress effects on neurodevelopment and (2) the transgenerational inheritance of stress-induced neural system alterations.

## 3. Prenatal Stress-Induced Neurodevelopmental Alterations as Environmental Adaptations

The following two studies investigated prenatal stress-induced neurodevelopmental alterations in rodents to illustrate how such alterations could be understood as environmental adaptations.

### 3.1. Adaptive Prenatal Alteration to the Postnatal Environment

First, an elegant series of studies by Kaiser and colleagues of the effects of prenatal chronic stress associated with social crowdedness in guinea pigs has shown that offspring exposed to a prenatal social crowdedness stress, in which dames were repeatedly placed in a cage with an exceeding number of mates during pregnancy, exhibited gender-specific behavioral and molecular changes [33–37]. Thus, in normal male and female animals, the expressions of androgen/estrogen receptors in limbic areas were high and low, respectively; however, these receptor expressions decreased in the male offspring, whereas the expressions increased in the female offspring among animals exposed to prenatal social crowdedness stress. Prenatal social crowdedness stress also caused behavioral alterations, such as more frequent observations of adult male offspring resting with body contact with cage mates, as well as decreased sexual motivation despite an increased nonsexually motivated (i.e., play) courtship behavior. Because these behaviors are usually observed in the juvenile period and wane by adulthood, the persistence of these behaviors in adult animals exposed to prenatal stress indicates that delayed maturation processes extended even into adulthood. On the other hand, female animals exposed to prenatal social crowdedness stress exhibited play behavior, which is a male-specific behavior and, therefore, unusual to be observed in females, in addition to male-like courtship behavior, indicating that the behaviors of such female animals exposed to prenatal stress become more like those of males.

These alterations caused by prenatal social crowdedness, which make male animals feminized and infantilized and female animals masculine, could be understood as adaption strategies against the specific environmental condition, that is, an overpopulated society, in which animals exposed to prenatal social crowdedness stress expect to spend their postnatal life. Thus, in an overly populated social group with a hierarchy, the best strategy for a male is to avoid fighting while there are many competitors and wait until the number of competitors eventually decreases, which is expected to occur given resource limitation, to gain a higher social rank within the society. In this context, infantilizing and feminine characteristics to avoid fighting would be the best adaptive strategy. In contrast, regardless of the crowdedness of the social group, a female needs to secure resources (housing/foods) to care for her children, for which the best adaptive strategy is to be a stronger competitor with male characteristics. Of particular importance are these behavioral changes caused by prenatal stress function as adaption strategies when the postnatal environment is consistent with the prenatal environment that caused the stress. However, these behavioral alterations caused by prenatal stress result in maladaptations or deficits if the postnatal environment is inconsistent with the prenatal environment, that is, a normal stress-free condition.

In fact, the idea that prenatal and postnatal environmental inconsistencies cause disease has been already proposed and is known as the Barker hypothesis, as well as a thrifty phenotype [38], although such an idea has not yet been applied to neurodevelopment processes and the pathogenesis of psychiatric disorders. The Barker hypothesis, as its name indicates, is coined by the UK medical researcher, David Barker, who found that there were associations between low birth weight and the increased risks of lifestyle diseases, such as obesity, diabetes, cancer, and cardiovascular diseases in adulthood. Such associations could be explained as follows: a baby may be born with a lower birth weight partly due to the insufficient nutrition of the mother during pregnancy. Consequently, such a baby adapts to a low nutrition condition by decreasing their metabolism to conserve energy. However, when such a baby with a low metabolic rate is raised with normal nutrition after birth, the child will continue to conserve energy, leading to lifestyle diseases in adulthood. Thus, the cause of such lifestyle diseases is a mismatch between the actual and expected environments that prenatal environmental “adaptation” has developed for.

### 3.2. Preparatory Prenatal Alteration for the Postnatal Environment

Another study illustrated the neurodevelopmental changes associated with the prenatal environment, which could be understood as adaptations to the effects of prenatal and postnatal nutrition restriction on cortical synaptic development.

Cortical synaptic development consists of two processes: synaptogenesis and synaptic pruning. Synaptogenesis starts during the prenatal period and continues after birth. Then, synaptic pruning is followed by the formation of a mature neural network. For instance, in the prefrontal cortex, synaptic development has been shown to continue until adulthood, with synaptogenesis continuing throughout the juvenile period and synaptic pruning taking place in adolescence [39]. Given that cortical synaptic development is a process involving both the prenatal and postnatal periods, this process is influenced by both the prenatal and the postnatal environments. Thus, consistency between the prenatal and the postnatal environments should be an important determining factor of cortical development.

Leuba and Rabinowicz have shown that the number of dendritic spines, where excitatory synaptic contacts are made [40], of pyramidal neurons in the visual cortex is lower in mice that are raised in undernutritional conditions compared to mice raised in normal nutrition conditions [41, 42]. However, when offspring born from dames that had a nutritional intake that was restricted during pregnancy are raised in an undernutritional condition, the number of dendritic spines in such offspring does not differ from that of normal mice, suggesting that the synaptic development alterations caused by prenatal undernutrition counteracts those caused by postnatal undernutrition. Thus, in this case, it appears that the alteration caused by a prenatal environment works as a preparation for the expected postnatal environment, through which the prenatal environment-associated alteration is normalized. Importantly, however, if a postnatal environment is not matched with the condition expected from the prenatal environment, such a prenatal environment-associated alteration results in maladaptation or a deficit.

We have recently investigated the effects of prenatal and postnatal restraint stress interactions on spatial memory in mice [43]. There have already been many studies showing that prenatal restraint stress causes spatial memory impairments [26]. In addition, adult rodents subjected to chronic restraint stress have similar, if not identical, spatial memory deficits [13]. However, it is still unknown how the prenatal and postnatal stress interaction (prenatal and postnatal environmental consistency) affects spatial memory. A preliminary result in our study indicates that when offspring born from dames exposed to prenatal restraint stress are exposed to 2 weeks of chronic restraint stress again during the juvenile period, these mice exhibit spatial memory comparable to that of normal mice, suggesting that matching between the prenatal and postnatal environments results in the normalization of the spatial memory function.

### 3.3. Two Types of Prenatal Alterations

Collectively, this evidence suggests that there are two different types of adaptation processes associated with prenatal environmental conditions. One is an “adaptive plasticity change” in which an alteration associated with a prenatal environment may work better in a postnatal environment if matched with the prenatal environment. The other is a “preparatory plasticity change,” in which an alteration associated with a prenatal environment may be normalized when the postnatal environment matches the prenatal environment.

Epidemiological studies have shown that adverse antenatal maternal environmental conditions, such as stress, virus infection, and malnutrition, during pregnancy increase the risks of the offspring developing psychiatric disorders, such as schizophrenia, MDD, ADHD, and ASD [7–11]. Considering that neurodevelopmental changes induced by prenatal environmental factors, such as stress, may be adaptions to the expected postnatal stressful environment, neurodevelopmental changes that increase the risks of psychiatric disorders could also be understood as environmental adaptation strategies against specific environments that are the sources of stress to organisms. Thus, some behavioral traits that are associated with psychiatric disorders could be understood to be either preparatory or adaptive changes to cope with expected postnatal environments.

Psychiatric symptoms that are already apparent in childhood, such as those of ADHD and ASD, may be understood to be adaptive changes, such that ADHD and ASD symptoms may be expressed regardless of the consistency between the prenatal and postnatal environmental conditions. Expressions of behavioral phenotypes associated with these disorders at early ages are crucially important for survival and reproductive success in animals in a wild environment and a hunter-gather society in case of humans. ADHD symptoms consist of hyperactivity, inattention, and impulsivity, all of which facilitate survival in a prey-predator interaction, with hyperactivity and inattention enabling exploration and scanning of predators in wider area and impulsivity leading to quick decision for escape from predators [44]. In addition, individuals with ASD have been suggested to have similar behavioral characteristics to those of animals [45], although whether cognitive processes are also similar between ASD individuals and animals is still under debate [46]. Thus, subjects with ASD symptoms are expected to have more successful survival and reproduction in wild environments than normal subjects. One example of such cases may be Victor of Aveyron, who was a boy found in the forests of Southern France more than 200 years ago. A description by the medical doctor of behavioral characteristics of this boy who was living in the wild environment has been suggested to resemble ASD symptoms [47–49].

There are also psychiatric conditions with delayed onsets, such as schizophrenia and MDD, which typically emerge in adolescence and adulthood and may be understood to be preparatory changes, such that expressions of the disorders are due to mismatches between the prenatal and postnatal environments. Expressions of symptoms of these disorders in a normal environment are mostly disadvantages for successful reproduction. In contrast, in an adverse environment, normal subjects have been shown to exhibit brain dysfunction [19]. Thus, any strategy that maintains brain function normal in such an adverse environment could yield a higher reproductive success. In this context, delayed expressions of symptoms of these psychiatric disorders in early adulthood are also advantageous for reproduction to determine whether these phenotypes should be maintained or eliminated, depending on whether an environment is adverse or normal at the time of reproduction.

## 4. Inheritance of Stress-Induced Alterations

Considering the evolutionary aspect of stress-induced behavioral and neuronal changes, the most important issue is that such stress-induced changes have to be inheritable. This issue is faced with two major problems. First, the inheritance of environmentally induced changes is not compatible with Darwinian evolution (natural selection) [50, 51]. In contrast, such inheritance is consistent with the evolutionary theory proposed by Lamarck [25], which has been rejected by the majority of evolutionary biologists. Second, the inheritance of deficits, which are disadvantageous for reproduction success, contradicts evolutionary theory, as evolution should be accompanied by successful reproduction. Regarding the later issue, as we have discussed in the previous section, there is accumulating evidence that leads us to suggest that stress-induced changes may not necessarily be deficits but could be advantageous adaptive strategies depending on the environmental context.

In fact, the finding that stress-induced behavioral changes could be inherited by descendants was previously reported in 1970 [52]. In this study, Wehmer and colleagues found that stress administered before and during pregnancy in dames caused behavioral alterations, such as heightened anxiety in the offspring. Moreover, such alterations were transgenerationally inherited into grandoffspring that did not experience stress at all. Even before this report, Waddington had also shown that environmentally induced changes are inheritable. Thus, he demonstrated that crossveinless induced by heat in fruit flies was inherited by descendants through an unidentified mechanism that incorporated the phenotypic change into a genetic mutation, which is now known as genetic assimilation [53]. More recently, instances of transgenerational inheritance of environmentally induced behavioral, physiological, and neuronal alterations in both animals and humans have been documented. A number of review articles of these findings are already available [22, 54–58].

Accumulating evidence suggests that epigenetic regulation of gene expression through mechanisms such as histone acetylation and DNA methylation is the molecular mechanisms that mediate the transgenerational inheritance of environmentally induced behavioral, physiological, and neuronal alterations [56, 59]. These biochemical modifications of DNA have been shown to be transmitted into descendants through paternal and/or maternal gametes [60]. Accordingly, the transgenerational inheritance of epigenetic-based alterations should involve gamete-specific gene expression, that is, genomic imprinting [60–62]. It is interesting to note that the genomic imprinting of several candidate genes associated with psychiatric disorders has been reported [63]. Most studies that have investigated transgenerational epigenetic inheritance in mammals follow its inheritance for a few generations; it remains unclear whether the biochemical processes, such as histone modification of chromatin structures and DNA methylation, could be maintained across additional generations. Nonetheless, transgenerational epigenetic inheritance has also been shown to play a significant role in the domestication of animals, such as the white leghorn, which is domesticated from a red jungle fowl [64], suggesting that transgenerational epigenetic inheritance could be a sustainable mechanism in many generations.

Psychiatric disorders have strong genetic backgrounds, including genetic mutations and variations (e.g., copy number variations and single nucleotide polymorphisms). Thus, even if the epigenetic bases of environmentally induced changes are transgenerational, they require a mechanism for translating epigenetic changes into equivalent genetic changes to demonstrate that this mechanism is involved in the causes of psychiatric disorders. One speculative explanation is that epigenetic processes may somehow promote genetic recombination, which in turn leads to genetic mutations and variants associated with psychiatric disorders. Such a link between epigenetic changes and genetic mutations may eventually prove that the mechanisms of the onset of psychiatric disorders often require gene x environment interactions [65, 66]. In this regard, psychiatric disorders may also involve mechanisms similar to those of phenotypic plasticity [67].

**Figure 1:**
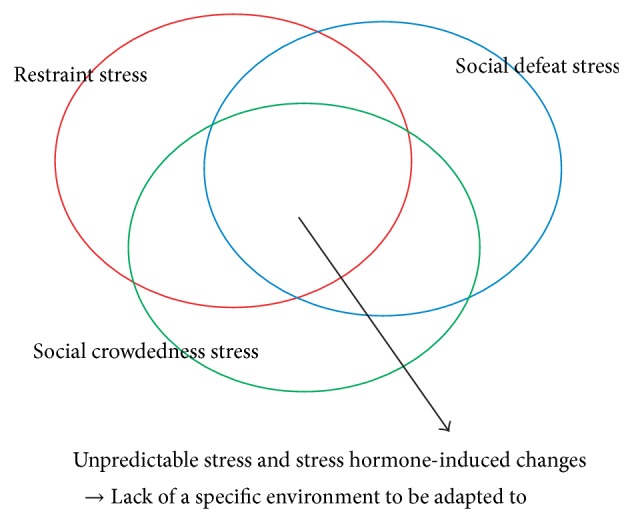
A schematic diagram illustrating that some stress-induced alterations may be heritable, but others may not be.

When considering the transgenerational inheritance of stress-induced behavioral and neuronal alterations, it is particularly important to note that stress-induced changes are not uniform but are variable and dependent on the environments that generate stress. This is supported by a number of findings, such as the fact that chronic restraint, but not unpredictable stress, causes heightened anxiety and remodeling of the neural network in the basolateral amygdala in a gender-specific manner [68, 69]. Moreover, prenatal restraint stress has been shown to cause a spatial memory impairment, whereas other prenatal stress procedures or prenatal administration of the synthetic stress hormone, dexamethasone, do not cause a spatial memory impairment in offspring [26]. Indeed, restraint stress is accompanied by the restriction of spatial information while animals are placed in restrainers. In animal studies, physical stress procedures, such as restraint, elevated platforms, cold, pain (e.g., foot-shock), and unpredictable (a mixture of different stressors administered each day) stress, have been frequently utilized. In addition, social stress procedures, such as social defeat, crowdedness, and isolation, have also been commonly employed. There has been no systematic investigation to compare the behavioral and neuronal alterations caused by different stressors, and, consequently, the studies that have investigated stress effects quite often assume that stress-induced alterations caused by different stressors are similar or identical. Indeed, stress may be identical in terms of an increase of stress hormones. However, such an increase of stress hormones could be a signal for adaptation, and how the system alters may depend on the specific stressful environment to which the system was exposed. Collectively, it is plausible that stress-induced changes may consist of heritable and nonheritable components, of which inheritable stress-induced changes may be understood to be environmental adaptation strategies (Figure 1). In this regard, unpredictable stress is remarkable in that the stressful environments change daily such that there is no particular environment for adaptation. This stress procedure has been frequently used in animal research, as habituation to stress environments can be minimized by this procedure. Thus, a comparison of the transgenerational inheritance of stress-induced changes between unpredictable stress and other stress procedures would be a promising direction of investigation.

## 5. Understanding Psychiatric Disorders as Environmental Adaptation

**Figure 2:**
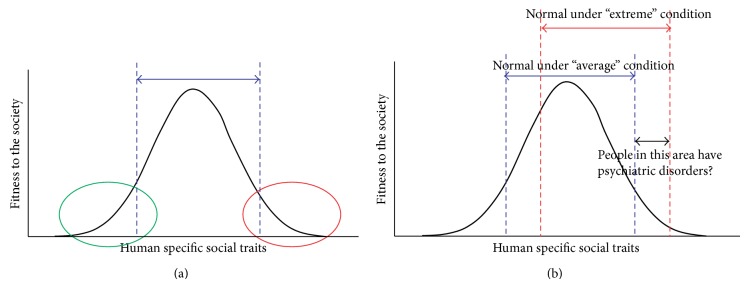
A schematic diagram illustrating that (a) psychiatric conditions may be understood to be the extreme ends of a normal distribution of brain function and (b) whether behavioral phenotypes associated with psychiatric disorders could be advantageous or disadvantageous may depend on the environment.

Based on the above discussions, psychiatric disorders may be understood to be an array of behavioral traits that have emerged as environmental adaptation against adverse stressful environments in the evolution of organisms. Thus, although such behavioral traits are considered maladaptive and disadvantageous in a “normal” environment, they may enable higher survival and reproductive rates in adverse environments. Thus, psychiatric disorders may have been present in humans and possibly other organisms as reservoirs to prevent the extinction of species against severe and stressful environments which endanger their survival and reproduction. This may be better intuitively understood by considering the model illustrated in Figure 2. When a specific behavioral trait or brain function is considered within a population, an exceeding or insufficient function distributed at the extreme ends of the distribution may correspond to psychiatric conditions in the normal environment (Figure 2(a)). On the other hand, in a severe and stressful environment, some populations with over- or underfunctioning in the distribution in the normal environment may no longer be considered abnormal (Figure 2(b)). These adaptive phenotypes associated with psychiatric disorders may not be selected over time and, therefore, may not become common traits among populations, since a severe, stressful event may not last for sufficiently long time or happen only in a microenvironment.

**Figure 3:**
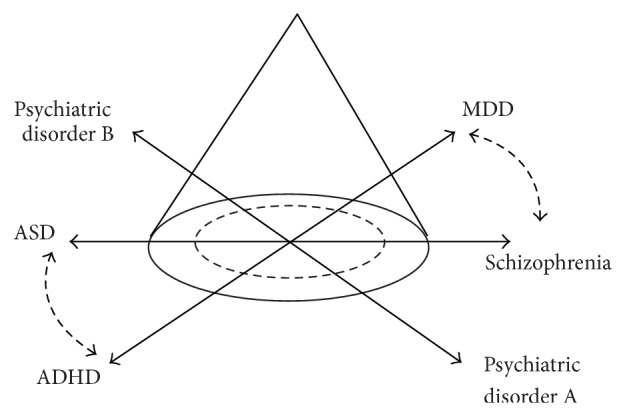
A schematic diagram illustrating that psychiatric disorders may be understood to be occurring on a spectrum with pairs of conditions with diametrical relationships.

For this model be true, there are at least two requirements. First, psychiatric disorders are continuous, but not discrete, deviants from normal conditions. Empirical evidence supports the continuous relationships of psychiatric symptoms, such as psychosis [70], externalizing behaviors [71], and autistic traits [72], with normal conditions. Second, if psychiatric disorders are the extreme ends of normality, there would always be one extreme end of a psychiatric condition that corresponds to the other extreme ends (e.g., hyper- versus hypofunction). Thus, there should be pairs of psychiatric conditions that have oppositional relationships with each other (Figure 3). In fact, there are several diagnostically distinct psychiatric disorders that appear to have such diametrical (oppositional) phenotypic relationships with each other. Crespi and colleagues have shown that schizophrenia and ASD are one such case of a diametrical relationship, as evidenced by the overlapping candidate genes suggested in both schizophrenia and ASD, although the copy number variations in several of these genes are opposite, with copy number expansions in one disorder and deletions in the other [73]. We have also recently suggested that MDD and ADHD may have some diametrical relationships in several of their phenotypes (psychomotor retardation versus hyperactivity, thought suppression versus impulsivity, and rumination versus inattention), which may be associated with hyper- and hypoactivity, respectively, of the habenula, one of the key brain regions regulating monoamine transmission in MDD and ADHD, respectively [74]. These arguments also inevitably predict that although, in the current clinical situation, each psychiatric disorder has been categorized, they may not be biologically discrete conditions but rather spectrums.

## 6. Conclusions

In this paper, we have explained, using the supporting literature, that there are three emerging psychiatric disorder issues that can be viewed from an evolutionary perspective. These issues are as follows: (1) some behavioral traits associated with psychiatric disorders may work beneficially in specific stressful environments that are otherwise difficult for organisms to survive and maintain reproductive success in; (2) behavioral and associated neuronal changes caused by prenatal stressful environments could be adaptive strategies against postnatal environments that are expected to be stressful; and (3) some, but not all, behavioral and associated neuronal changes caused by prenatal and postnatal stressful environments could be inheritable.

Collectively, a hypothesis that psychiatric disorders may have emerged as adaptation strategies against adverse environmental conditions in the evolution of organisms is formulated. Thus, psychiatric conditions may be a strategy for creating biodiversity to maintain the species in case unusual environmental changes occur. This idea, however, still has major drawbacks. For instance, although psychiatric disorders involve genetic mutations, no empirical evidence to date has documented the mechanisms that translate epigenetic changes into equivalent genetic mutations. Nevertheless, our hypothesis may also yield several novel insights into our understanding of psychiatric disorders, such as (1) why stress is often associated with the onset, relapse, and exacerbation of symptoms in several psychiatric disorders; (2) why the onset of symptoms in some psychiatric disorders is apparent early in development, whereas others have delayed onset in adulthood; (3) why psychiatric disorders may not be discrete conditions from the normal but continuous extremes; and (4) why symptoms of different psychiatric disorders may have oppositional relationships, which in turn suggests that psychiatric disorders could exist on a spectrum and are not categorical.

Indeed, the concept proposed here is highly speculative at this moment, and investigations to validate, modify, or reject the concept have been awaited. For instance, higher incidence of schizophrenia has been shown to be associated with urbanicity [75, 76]. Although which aspects of environmental factors in the urbanicity are involved and whether either urban birth or urban residence or both of them are involved in such higher prevalence of the disorder have been still unclear, this observation appears to be somewhat controversial to the proposed concept. Further epidemiological studies such as those investigating an incident rate of schizophrenia in subjects born in an urban city and upbringing in a rural area and vice versa would yield more insights on this issue. Moreover, another major limitation of the concept also includes how these environmental adaptation processes could interact with genetic predispositions of psychiatric disorders which has also remained unclear at this moment. Future investigations of the molecular and neural network changes associated with stress under evolutionary perspectives would be able to further our understanding of the biological mechanisms and therapeutic treatments of psychiatric disorders.
